# *FGFR1* Amplification and Response to Neoadjuvant Anti-HER2 Treatment in Early HER2-Positive Breast Cancer

**DOI:** 10.3390/pharmaceutics14020242

**Published:** 2022-01-20

**Authors:** María Gaibar, Apolonia Novillo, Alicia Romero-Lorca, Diego Malón, Beatriz Antón, Amalia Moreno, Ana Fernández-Santander

**Affiliations:** 1 Health Sciences Faculty, Universidad Francisco de Vitoria, 28223 Madrid, Spain; maria.gaibar@ufv.es; 2 Biomedical and Health Sciences Faculty, Universidad Europea de Madrid, 28670 Madrid, Spain; apolonia.novillo@universidadeuropea.es (A.N.); alicia.romero@universidadeuropea.es (A.R.-L.); 3 Department of Oncology, University Hospital of Fuenlabrada, 28942 Madrid, Spain; diego.malon@salud.madrid.org (D.M.); beatriz.anton@salud.madrid.org (B.A.); amorenot@salud.madrid.org (A.M.)

**Keywords:** HER2-positive breast cancer, anti-HER2 treatment, *FGFR1* gene, CNVs, pathological complete response, Miller–Payne grading

## Abstract

HER2-positive breast cancer (BC) is an aggressive subtype that affects 20–25% of BC patients. For these patients, neoadjuvant therapy is a good option that targets a pathological complete response (pCR) and more breast-conserving surgery. In effect, the outcomes of patients with HER2-positive BC have dramatically improved since the introduction of anti-HER2 antibodies such as trastuzumab (TZ) and/or pertuzumab (PZ) added to chemotherapy. This study sought to examine whether correlation exists between copy number variations (CNVs) in several genes related to the PI3K/AKT pathway (*HER2, FGFR1, PIK3CA, AKT3* and *MDM2*) and the efficacy of anti-HER2 neoadjuvant treatment in patients with early HER2-positive BC. Forty-nine patients received TZ or PZ/TZ and chemotherapy as neoadjuvant treatment. Gene CNVs were determined by quantitative polymerase chain reaction on paraffin-embedded biopsy specimens. The response to 6 months of therapy was assessed by Miller–Payne grading of the tumor on surgical resection; grades 4 and 5, indicating >90% tumor reduction, were defined as a good response. A good response was shown by 64.5% and a pCR by 31.2% of patients. When stratified by anti-HER2 antibody received and gene CNV, it was found that patients with *FGFR1* gene amplification or those with *FGFR1* amplification treated with TZ alone showed a poor response (*p* = 0.024 and *p* = 0.037, respectively). In the subset of patients treated with TZ/PZ combined, the pCR rate was significantly lower among those showing *FGFR1* amplification (*p* = 0.021). Although based on a small sample size, our findings suggest that patients with *FGFR1* amplification might benefit less from anti-HER2 antibody therapy.

## 1. Introduction

The human epidermal growth factor receptor 2 (*HER2* or *ERBB2*) gene occurs in the 17q21 chromosome region and encodes a transmembrane glycoprotein with tyrosine kinase activity. HER2 is involved in the regulation of cell growth, differentiation, and invasion and it is well known that its overexpression is associated with an aggressive phenotype and poor prognosis in breast cancer (BC) patients [1]. The frequency of this HER2-positive phenotype BC is between 20 and 30% of all BC [2]. Neoadjuvant therapy is a good option for operable early BC and targets a pathological complete response (pCR) detected upon surgery. Besides improving the chances of operability and offering more breast-conserving surgery, neoadjuvant treatment has been reported to improve disease-free survival and overall survival in patients with early stage HER2-positive BC [3].

Trastuzumab (TZ) is the first humanized anti-HER2 monoclonal antibody that acts against the extracellular domain of the human HER2 protein. This antibody inhibits the ligand-independent HER2 and HER3 signaling through the PI3K/AKT pathway (phosphoinositide-3-kinase/Akt serine/threonine kinase) that induces antibody-dependent cellular cytotoxicity [4]. Since the introduction of TZ added to chemotherapy (CT) nearly two decades ago, the outcomes of patients with HER2-positive BC have dramatically improved. In the NOAH trial, addition of TZ to the treatment regimen for women with locally advanced BC both increased the pCR rate from 19% to 38% and led to a significant improvement in event-free survival [5]. Pertuzumab (PZ) is a monoclonal antibody directed against the dimerization domain of HER2 and also works against the HER2 pathway. Dual HER2 blockade induced by adding PZ to TZ in neoadjuvant CT has been shown to increase the pCR rate compared with TZ alone, giving rise to a rate of around 50–70% [6].

The PI3K/AKT pathway can be activated by different signaling molecules apart from ligand-independent HER2 and HER3 signaling or HER2 dimerization. FGFR1 is a member of the FGFR family that exhibits a highly conserved structure among different receptors of fibroblast growth factors. The interaction between fibroblast growth factors and FGFRs has been related to cell differentiation, proliferation, and survival [7]. FGFR and EGFR signaling may mediate the downstream phosphoinositide-3-kinase/Akt serine/threonine kinase (PI3K/AKT) pathway. Akt mediates the negative control of p53 levels through phosphorylation of MDM2, which is necessary for p53 degradation [8]. The activation of MDM2 by Akt and reduction in p53 levels has also been observed in HER2-overexpressing BC cells, with the consequence of promoting tumor progression [9]. *FGFR1* gene amplification was observed in 8–15% of all cases of BC [10], and results in the aberrant activation of the downstream PI3K/AKT pathway, promoting cell cycle progression and apoptosis inhibition. 

Breast cancer is a very heterogeneous disease with many different polymorphisms detected among BC patients. In the past few years, frequent gene copy number variations (CNVs) have been identified, such as those affecting the genes *FGFR1, HER1, HER2* or *PIK3CA*, involved in the PI3K/AKT pathway. In effect, CNVs in a wide array of these genes have been attributed an important role in the variability of clinical outcomes [11]. 

The response to anti-HER2 neoadjuvant treatments for HER2-positive BC varies widely among patients. For instance, ~50% of patients were found not to benefit from TZ neoadjuvant treatment [12]. Thus, it is extremely important to distinguish between HER2-positive breast cancer patients who are more likely to respond well to anti-HER2 treatment and those who are not. The aim of this study was to examine the possible association between CNVs in five genes related to the PI3K/AKT pathway, *HER2, FGFR1, PIK3CA, AKT3* and *MDM2*, and the efficacy of anti-HER2 blockade neoadjuvant treatment in patients with early HER2-positive BC.

## 2. Materials and Methods

### 2.1. Design and Patients

Forty-nine female patients diagnosed with HER2+ breast cancer were treated at the Oncology Department of the University Hospital of Fuenlabrada, Madrid, Spain, between 2010 and 2018. In each patient, HER2/ER/PR status was determined as part of the routine diagnostic procedure. Patients received TZ or PZ/TZ combo and CT as neoadjuvant therapy to allow for breast-conserving surgery and a pathological complete response. The therapeutic regimens and targeted therapy used were based on current standard of care in each case: (i) anthracycline-based therapy (epirubicin, cyclophosphamide with weekly paclitaxel), (ii) carboplatin-docetaxel therapy, or (iii) taxane monotherapy (weekly paclitaxel). Patient samples and data were collected by medical oncologists at the Oncology Department, in accordance with the Declaration of Helsinki. The study protocol was approved by the Hospital’s Ethics Committee (identification code: APR 18/20, September 2018). Informed consent for the experimental use of biopsies/tumors collected for diagnosis was provided by all patients.

Response to treatment was assessed using the Miller–Payne tumor grading system (1 = no tumor reduction, 2 = up to 30% tumor reduction, 3 = 30–90% tumor reduction, 4 = >90% tumor reduction (considered close to a pCR), and 5 = no invasive malignant cells identifiable in sections from the tumor site (considered a pCR)). For the present purpose, patients showing Miller–Payne grades 4 and 5 were defined as good responders and those with grades 1 to 3 were classified as poor responders. Tumor response grade was assessed after 6 months of treatment at surgery resection. 

Overall survival (OS) was determined based on the time from treatment onset to the date of any-cause death or last follow-up.

### 2.2. CNV Selection and Analysis

The five CNVs examined here were selected on the basis of published evidence of somatic alterations in specific genes identified in HER2-positive breast cancer patients (see for instance [13]). The locations of the studied CNVs in the genes *HER2, FGFR1, PIK3CA, AKT3* and *MDM2* are provided in Table 1. The five genes selected are those involved in the HER2 pathway with an important role in patients showing HER2 overexpression such as the present participants. HER2 and FGFR1 receptors act upon the PI3K/AKT intracellular signaling pathway, and play a key role in cell cycle regulation [14]. AKT phosphorylates and activates MDM2 protein, an important negative regulator of p53. 

Total DNA was extracted from 2.5 mm3 of paraffin-embedded tumor biopsies, as required by the QIAamp^®^ DNA FFPE Tissue Kit (Qiagen, Germantown, MD, USA). Each 10 µL reaction mixture contained 5 ng of gDNA as the PCR template, 1× TaqMan Gene Expression PCR Master Mix (5 µL) along with 1× TaqMan™ Copy Number Assay (Hs00709630_cn, Hs06659652_cn, Hs05793001_cn, Hs03082319_cn, Hs00770365_cn, Table 1) and 1× TaqMan™ Copy Number Reference Assay (RNase *p*). Real-time PCR plates were run in an QuantStudio 12K Flex Real-Time PCR System (Applied Biosystems, Thermo Fisher Scientific, Waltham, MA, USA) under standard running conditions (95 °C for 10 min and 40 two-step cycles consisting of 95 °C for 15 s and 60 °C for 1 min) and analyzed with Copy Caller Software (Applied Biosystems, Thermo Fisher Scientific, Waltham, MA, USA). All reactions were performed in quadruplicate.

### 2.3. Statistical Analysis

Quantitative variables are provided as medians and their interquartile range or means and their standard deviation depending on their distribution (Shapiro–Wilk test for normality). For qualitative variables, absolute and relative frequencies are given as percentages. The relationship between each gene’s CNV and tumor response (good and poor response) was assessed using the Mann–Whitney or Student *t* tests according to the normality of the data. In patients stratified according to the anti-HER2 treatment received, the Chi-square test was used to compare rates of good responders to each treatment. Time-to-event data were analyzed using the Kaplan–Meier method. All calculations were performed using the Program Stata v.14.2 (Stata Corp, LLC, Lakeway, TX, USA). Significance was set at *p* ≤ 0.05.

## 3. Results

### 3.1. Patient Characteristics

Table 2 shows the clinical characteristics of the BC patients enrolled. All of them were women (100%) of median age 50 years (range 28.4–74.6 years). Tumor locations were 42.9% right side and 57.1% left side. Most tumors were ductal, 93.9% compared to 6.1% for lobular. Histology grades on diagnosis were 14.3%, 53.0% and 32.7%, for grades 1, 2, and 3, respectively. All patients were positive for HER2, ER and PR and most had no metastasis on diagnosis (95.9%). The anti-HER2 drug received was TZ in 65.3% of the patients and TZ + PZ combo in the remaining 34.7%. Chemotherapy regimens based on current standard guides were anthracycline-based therapy in 22.5%, carboplatin-docetaxel therapy in 61.2% and taxane monotherapy in 16.3% (Table 2).

After six months of therapy with TZ or PZ/TZ and CT, 64.5% of the BC patients showed a good response as indicated by >90% tumor reduction (Miller–Payne grade 4 + 5). A pathological complete response was observed in 31.2% of patients (Table 2). For one patient, the Miller–Payne response was unknown. After stratifying patients according to the anti-HER2 treatment received, rates of good responders were similar in the patients treated with TZ (58.1%) versus TZ/PZ (76.5%) (*p* = 0.202). No correlation was detected between response to therapy and the clinical characteristics of the patients.

### 3.2. Gene Amplification in Relation to Treatment Effectiveness and Survival

Of the five genes examined, amplification of the FGFR1 gene was significantly associated with the response shown to the anti-HER2 drugs. Hence, patients showing a poor tumor response (Miller–Payne grades 1–3) had significantly more FGFR1 gene amplification (*p* = 0.024, Table 3). More FGFR1 amplification was also associated with a poor response in the subgroup of patients treated with TZ alone (*p* = 0.037). Furthermore, within the TZ/PZ combo group, the pCR rate was significantly lower in patients showing greater FGFR1 gene amplification (*p* = 0.021). 

Five-year survival was 87.02% (95% CI: 68.98–95.95); only four patients died at 50 to 65 months. Because of the low mortality, median survival could not be determined, and it was not possible to assess the relationship between each CNV and survival.

## 4. Discussion

The neoadjuvant approach is a standard therapy option for operable early breast cancer, and has been associated with a very good pCR at the time of surgery. According to the results of Collaborative Trials in Neoadjuvant Breast Cancer (CTNeoBC) therapy in HER2-positive BC patients, women showing a pCR have a significantly better overall survival rate than those almost showing a pCR [3,5]. This has meant that a pathological complete response is regarded as an informative biomarker of survival to be considered in the approval of new treatments by the EMA (European Medicines Agency) [15]. Both the addition of TZ to neoadjuvant treatment in early BC patients and dual HER2 blockade with TZ + PZ have led to significantly increased pCR rates [5,6]. In our study, BC patients showed a good response rate (Miller Payne grades 4–5) both to TZ therapy and dual TZ + PZ HER2 blockade (58.1% and 76.5%, respectively). Other authors have reported similar outcomes in larger patient series and concluded that neoadjuvant chemotherapy with TZ + PZ increases the pCR rate compared with trastuzumab alone to one of around 50–70% [6]. However, it should be mentioned that approximately one-third of patients with HER2-positive early BC treated with TZ experience relapse [16]. This highlights a need to find good biomarkers of the efficacy of anti-HER2 treatment for this aggressive BC phenotype. 

Mutations in genes with a role in the PI3K/AKT pathway have been examined in order to select patients who might benefit from anti-HER2 therapy, especially the dual blockade approach [1]. For instance, Han et al. suggested that HER2-positive early BC patients with a Val variant in the *HER2Ile655Val* SNP had a better survival when treated with TZ versus patients not receiving TZ, although the typed mutation itself would confer a more aggressive phenotype [17]. Other studies have revealed that the presence of mutations in the *PIK3* gene, seen in 12 to 39% of HER2-positive BC patients, can lead to resistance to trastuzumab therapy, showing a lower pCR rate and shorter disease-free survival [18]. The genes examined in our study—*HER2, FGFR1, PIK3CA, AKT3* and *MDM2*—were selected on the grounds of their implication in this metabolic pathway and their known consequences on cell cycle regulation via inhibition of p53 [8]. Our results highlight that an amplified number of *FGFR1* gene copies seems overall related to a significantly worse efficacy of treatment (*p* = 0.024, Table 3). Besides the whole patient sample, this poor response was also observed in the subgroup of patients treated only with TZ (*p* = 0.037) but not in the subgroup given the TZ + PZ combo. The small sample size of both subgroups is a limitation of our study, preventing the observation of possible significant differences and multivariate analysis to check for interactions between CNV and other data such as histological stage, as performed by Fountzilas et al. with *TP53* and *PIK3CA* mutation status [19]. Nevertheless, in women treated with TZ + PZ, having an amplified *FGFR1* gene was effectively linked to a significantly lower pCR (Miller–Payne grade 5, *p* = 0.021).

Amplification of the *FGFR1* gene is its most common variation, occurring in 8–15% of all cases of breast cancer [20]. *FGFR1* amplification has been considered a marker of resistance of cell lines to endocrine therapy in in vitro studies [21]. Our patients who were poor responders had both amplified *FGFR1* and *HER2* as all participants were HER2-positive at diagnosis (as an inclusion criterion). According to Chen et al., patients with *FGFR1* and *HER1/2* co-amplification exhibit a less favorable prognosis compared with patients with either *FGFR1*, *HER1/2* amplification or with no amplification of these genes [14]. However, other studies have related *FGFR1* amplification to an improved response of BC patients to other treatments. For instance, Hui et al. concluded that HR-/HER2-negative metastatic BC patients showed an apparent increase in overall response to lucitanib if they had a high number of *FGFR1* copies [22]. Also seemingly relevant is the link detected between PI3K/AKT pathway activation and both worse trastuzumab efficacy and median time to progression and survival [23]. These last authors also reported an increased risk of disease progression in HER2-positive BC patients with mutations in the *PIK3CA* gene [23].

Several clinical trials have shown that TZ can significantly increase disease-free survival and overall survival in HER2-positive BC patients in both metastatic and early-stage settings (see for instance, [24]). The five-year survival rate in our study was 81.6%, and only four patients died. Due to this low mortality, median survival could not be measured, and it was not possible to assess the link between each CNV and survival.

Hanker et al. described that the FGFR and HER2 pathways can cooperate to promote tumor progression as a result of somatic mutations in genes related to the former pathway that could play a role in resistance to anti-HER2 drugs in HER2-positive BC tumors [25]. These authors considered that patients showing *FGFR1* expression in the bottom 80% percentile had a significantly reduced risk of disease recurrence when treated with TZ. However, TZ did not reduce the risk of recurrence in the top 20% *FGFR1* expression percentile. Based on the results of in vitro and in vivo mice studies, Akhand et al. suggested that enhanced *FGFR1* expression also confers resistance to anti-HER2 antibody therapies by disrupting trastuzumab’s ability to bind to HER2 [26]. Although the mechanisms whereby FGFR prevents trastuzumab binding have not yet been determined, recent studies have unveiled the relevant role of micro-RNAs in gastric cancer HER2-positive cells. Guo et al. proposed that miR-301a-3p disseminated TZ resistance by activating the expression of *FGFR1* through the intercellular transfer of this micro-RNA by exosomes [27]. 

The field of anti-cancer drug development is presently focused on the identification of biomarkers for more personalized treatment. In effect, CNVs have shown their potential utility for predicting the risk of disease progression in patients with solid tumors [28]. Our study in patients overexpressing the *HER2* gene (HER2-positive) shows that *FGFR1* gene amplification is associated with a poor tumor response to both anti-HER2 treatments, trastuzumab and trastuzumab plus pertuzumab. Although the sample size is a limitation, because of our results do not reach the minimum accepted 80% statistical power, the findings of this study provide direction for future studies designed to address this relationship. Future clinical trials should also explore whether resistance to anti-HER2 therapies could be diminished by HER2 and FGFR inhibitor drug combinations.

## Figures and Tables

**Table 1:** Details of the assays and genes used in the detection of CNVs.

Gene	Thermo Fischer ID	Target Size (bp)	Location on GRCh38 (Cytoband)	Gene Location Identified
*FGFR1* (fibroblast growth factor receptor 1)	Hs00770365_cn	75	Chr.8: 38457439 (8p11.23)	within exon 5
*HER2* (ERRB2 receptor tyrosine kinase 2)	Hs00709630_cn	101	Chr.17:39723405 (17q12)	within exon 22
*MDM2* (MDM2 proto-oncogene)	Hs03082319_cn	99	Chr.12: 68835830 (12q15)	overlapping intron 10—exon 11
*PIK3CA* (phosphatidylinositol-4,5-bisphosphate 3-kinase catalytic subunit alpha)	Hs06659652_cn	77	Chr.3: 179155387 (3q26.32)	within intron 3
*AKT3* (serine/threonine kinase 3)	Hs05793001_cn	100	Chr.1: 243806024 (1q44)	within intron 4

**Table 2:** Baseline characteristics of the 49 patients enrolled in this study.

Patient Characteristics	No. (%)
Median age (years)	50.0, range 28.4–74.6
Gender - Female - Male	49 (100) --
Tumor location - Right-sided - Left-sided	21 (42.9) 28 (57.1)
Histology type - Ductal - Lobular	46 (93.9) 3 (6.1)
Histology grade - Grade 1 - Grade 2 - Grade 3	7 (14.3) 26 (53.0) 16 (32.7)
HER2 status - Positive - Negative	49 (100) --
ER status - Positive - Negative	49 (100) --
PR status - Positive - Negative	49 (100) --
Metastasis - No - Yes	47 (95.9) 2 (4.1)
Miller–Payne response grade - 1 - 2 - 3 - 4 - 5	1 (2.1) 7 (14.6) 9 (18.8) 16 (33.3) 15 (31.2)
Anti-HER2 drug - Trastuzumab - Trastuzumab + pertuzumab	32 (65.3) 17 (34.7)
Chemotherapy - Anthracycline-based therapy - Carboplatin-docetaxel therapy - Taxane monotherapy	11 (22.5) 30 (61.2) 8 (16.3)

**Table 3:** Association between gene CNVs and anti-HER2 treatment response (Miller Payne 1-3 = poor response, Miller Payne 4-5 = good response).

Gene	Response	Mean	SD	Median	IQR	Range	*p* Value
*HER2*	Poor n = 17	3.72	5.00	2.00	2.42	0.78–21.63	0.464
	Good n = 31	3.75	3.14	3.03	3.82	0.27–12.19	
*FGFR1*	Poor n = 17	3.54	3.72	2.33	1.31	1.27–16.90	0.024
	Good n = 31	2.26	1.38	1.91	0.87	1.07–8.98	
*PIK3CA*	Poor n = 17	1.86	0.45	1.97	0.58	0.81–2.72	0.483
	Good n = 31	2.16	0.84	1.99	0.89	1.15–4.68	
*AKT3*	Poor n = 17	2.24	0.65	2.30	0.69	0.88–3.73	0.822
	Good n = 31	2.19	0.93	2.01	1.12	0.51–4.02	
*MDM2*	Poor n = 17	3.64	4.55	2.37	1.04	0.90–20.62	0.296
	Good n = 31	2.72	2.95	2.00	1.51	0.68–17.56	

## Data Availability

All data available are reported in the article.
